# A study of the immediate effects of glycerine-filled insoles, contoured prefabricated orthoses and flat insoles on single-leg balance, gait patterns and perceived comfort in healthy adults

**DOI:** 10.1186/s13047-015-0107-4

**Published:** 2015-09-07

**Authors:** Anna L. Hatton, François Hug, Brooke C. M. Brown, Leon P. Green, Jacob R. Hughes, Jarrad King, Emma J. Orgar, Kate Surman, Bill Vicenzino

**Affiliations:** School of Health and Rehabilitation Sciences, Therapies Building (84A), The University of Queensland, Brisbane, QLD 4072 Australia; Laboratory “Movement, Interaction, Performance” (EA 4334), University of Nantes, Nantes, France

**Keywords:** Shoe insoles, Foot orthoses, Single-leg balance, Gait, Comfort

## Abstract

**Background:**

Footwear interventions are often prescribed to assist with the management of lower limb pain, injury and disease. Commercially available shoe insoles and orthoses are increasingly incorporating novel design features to alleviate foot and lower limb symptoms, but this may be at a cost to optimal functional performance. This study compared the immediate effects of wearing glycerine-filled insoles, contoured prefabricated orthoses, and flat insoles, on balance and gait measures.

**Methods:**

Thirty healthy adults (17 men, 13 women; mean [SD] age: 24.3 [2.5] years) performed tests of single-leg standing with eyes open (Kistler force platform), star excursion balance test, and level-ground walking (GAITRite® walkway system), under three randomised conditions: wearing glycerine-filled insoles, prefabricated orthoses, and flat (control) insoles, within their own footwear. Centre of pressure movement (anterior-posterior and mediolateral range and standard deviation, total path velocity), star excursion balance test reach distance, and temporospatial gait variables were collected. Perceived comfort of the inserts was scored immediately after use on a 100 mm visual analogue rating scale. After trialling all inserts each participant ranked their level of comfort from least to most.

**Results:**

Centre of pressure measures, star excursion balance test reach distance, or temporospatial gait variables did not differ between the three inserts (all *P* values >0.088). Significant between-condition differences were reported for comfort ranking (*P* = 0.031), but not rating scores (*P* = 0.638). Weak to moderate negative correlations (r values ranged between −0.368 and −0.406) were observed between visual analogue scale comfort rating for the flat insoles and prefabricated orthoses, star excursion balance test and gait measures.

**Conclusions:**

Single-leg standing balance, star excursion balance test performance, and level-ground walking patterns in asymptomatic adults do not appear to differ when wearing glycerine-filled insoles, contoured prefabricated orthoses, or flat insoles. Perceived comfort may be related to the biomechanical or clinical effectiveness of novel footwear interventions, and requires further investigation. Importantly, these findings are specific to a healthy population and further research is needed to determine the long-term effects of glycerine-filled insoles in patients with known balance impairments.

## Background

Footwear interventions, such as shoe insoles and foot orthoses, are becoming increasingly popular in clinical and healthy populations. This is because they offer a simple, cheap, non-invasive intervention that may help to prevent lower limb overuse injuries [1], alleviate pain [2, 3], and maintain skin integrity at the feet [4]. Proposed underlying mechanisms by which footwear interventions may bring about their effects include three core paradigms: kinematic responses, shock attenuation, and alterations in sensorimotor control, or a combination of all [5, 6]. Traditional understanding of the role of foot orthoses focuses on their potential to provide mechanical support and biomechanical realignment, thereafter alleviating debilitating symptoms such as pain, or enhancing movement [5, 7]. Foot orthoses and insoles may also assist with prevention of ulceration by reducing shearing forces, dispersing plantar pressures, and providing shock absorption [5].

A wide variety of foot orthoses and insoles are emerging, which incorporate novel design features such as ridges of elastomeric material [8, 9], vibrating components [10, 11], and textured upper surfaces [12–16]. The latter claim to optimise performance by targeting sensorimotor mechanisms, such as stimulation of sensory receptors [17]. Glycerine-filled shoe insoles (BestSole Inc.) have recently been developed to alleviate lower limb pain, by way of providing a massaging effect to the plantar surface of the foot, and as such, may function in a similar manner to other interventions designed to enhance sensory information. The insoles contain 100 % fluid glycerine which flows from areas of high to low pressure, through cavities extending from the forefoot to heel regions, as individuals move. In line with current evidence, foot orthoses [18], insoles [19, 20], or floor surfaces [21] constructed from soft, compliant materials, have the potential to significantly attenuate plantar pressures and shear forces: which are common sources of lower limb pain and precursors to ulceration. This same design feature also appears to reduce mechanical support to the foot and ankle [22, 23], dampen awareness of foot position [24], and creates an unstable supporting surface which may be detrimental to balance and gait [17, 25]. Whilst footwear interventions constructed from low-density materials, may provide symptom relief, this may be at a cost to functional performance. Soft or compliant insoles could create a potentially unstable interface between the foot and ground, which may disrupt static or dynamic balance. Given the rapid growth of footwear industry, this is becoming an increasingly important area to research.

The primary aim of this study was to compare the immediate effects of wearing novel glycerine-filled shoe insoles, contoured prefabricated orthoses, and a flat (control) insole in healthy adults on: centre of pressure (CoP) movement during single-leg standing; star excursion balance test (SEBT) performance and; temporospatial gait parameters during level ground walking. Due to the viscosity of contents within the glycerine-filled insoles, we hypothesised that wearing this particular insert would lead to increased CoP movement during single-leg standing, reduced SEBT reach distance, and alterations in temporospatial gait measures that represent a more cautious walking pattern, relative to the orthoses and flat insole. Secondary aims were to explore any differences in perceived comfort between the three inserts, and to determine whether comfort was associated with measures of static and dynamic balance.

## Methods

### Participants

Thirty healthy adults (17 men, 13 women) with a mean (SD): age, 24.3 (2.5) years; height, 178.3 (10.0) cm; and body mass, 72.4 (12.4) kg, were recruited from the university staff and student base, in response to advertisements. All individuals who were willing to wear footwear interventions for the duration of the test procedures were included. Exclusion criteria were current use of shoe insoles or foot orthoses; history of lower limb or foot injuries, including pain, within the previous 12 months; any circulatory or sensory conditions affecting the feet or lower limbs; inability to walk 10 m unassisted; any other musculoskeletal, sensory or neurological impairments which are known to alter balance or gait performance; unable to understand or communicate in English and thus provide informed consent and follow instructions. Ethical approval was granted by the Medical Research Ethics Committee at The University of Queensland (#2014000140). Written informed consent was obtained from all participants. Standing foot positioning was quantified using the 6-item Foot Posture Index (FPI) [26] to characterise the study sample. Mean (SD) FPI score for the test leg was 4.6 (3.3). Normal foot posture (FPI score: 0 to +5) was observed in 66.7 % of participants (*N* = 20). 26.7 % of the sample (*N* = 8) presented with pronation (FPI score: +6 to +9), with the remaining 6.6 % (*N* = 2) reported to be highly pronated (FPI score: 10+).

### Procedures

All test procedures were conducted in a University laboratory. Using a within-subject repeated measures study design, balance and gait tasks were assessed for all participants during three different randomised shoe inserts. The order in which the SEBT and level-ground walking tests were performed was also randomised, with single-leg standing completed last due to laboratory constraints. Randomisation schedules were generated using an online list randomiser (www.random.org/lists/). To allow for familiarisation with the test procedures, all participants completed 1–2 practice trials of each balance and gait task prior to data collection.

### Single-leg standing with eyes open (StandEO)

StandEO tests were performed on either the right (*N* = 14) or left leg (*N* = 16) (randomly presented). As our participants were healthy, and did not present with unilateral lower limb impairments, we assumed that their response to shoe insoles and foot orthoses would be comparable between-legs. Participants were instructed to stand in the centre of a force platform on their randomly allocated test leg, with their opposite leg flexed to ~90° at the knee, with the legs not touching and arms folded across their chest [27], over 30 s. Participants were asked to look straight ahead at a black, circular, 15 cm diameter, visual target, positioned at eye level, located 3 m from the centre of the force platform. When a participant touched down with their raised foot, this was considered to be a balance failure and the test was discarded and repeated. For each insert, three StandEO repetitions were performed, and the average of each was calculated. A 10 s rest period was given between repetitions to prevent fatigue.

### Star excursion balance test (SEBT)

The SEBT is a reliable assessment of dynamic stability [28, 29] which has been used in previous studies to explore the effects of foot orthoses on balance performance [30]. Participants stood with either their right (*N* = 14) or left (*N* = 16) heel (randomly presented) in the centre of an 8-point star marked on the floor with tape (with each point set at a 45° angle), with their arms folded across their chest as described by Kinzey et al. [28]. All participants were given the standardised instruction to “*Reach as far as you can along the line without moving your standing foot, keeping your heel down. When touching the line with your reaching foot, try not to step or place all your weight down: lightly touch the ground then return to the starting position”.* Three repetitions were performed along each of eight directional lines: anterior, anteromedial, medial, posteromedial, posterior, posterolateral, lateral and anterolateral. All participants began the test by reaching in the anterior direction, and were thereafter randomised to completing the remaining reaches in either a clockwise (*N* = 16) or anticlockwise (*N* = 14) sequence. A marker was placed on the floor at the point where the most distal aspect of the reaching foot touched the ground. The distance from the centre of the star to the marker was determined using a tape measure, and averaged across the three repetitions for each direction. All participants were given a 10 s rest period between repetitions. Tests were discarded and repeated if a participant lost their balance, touched outside the directional lines, or bore weight through their reach foot.

### Level-ground walking

Temporospatial gait parameters were measured during level ground walking, at a comfortable self-selected speed in each of the three insert conditions, using an instrumented walkway system (GAITRite®, CIR Systems, Inc., Havertown, PA 19083, USA). The GAITRite® system is an electronic walkway, approximately 8.2 m long, with the active area being 0.61 m wide and 7.32 m long. The GAITRite® system is a reliable and valid tool for measuring gait performance in healthy adults [31]. To allow for acceleration and deceleration at the beginning and end of each gait trial, a taped line was placed 2 metres before and 2 metres after the GAITRite® walkway. Participants were positioned at the start line and given the following standardised instructions: “S*tarting at the blue line, walk along the length of the walkway at your usual, comfortable speed and continue until you pass the second blue line at the end of the walkway”.* For each temporospatial measure, the average of three ~12 m gait trials was calculated for each insert.

### Shoe inserts

The glycerine-filled shoe insoles (BestSole Inc., Boynton Beach, FL 33424, USA) were ~2 mm thick (at their most consistent thickness), composed of ethylene-vinyl acetate plastic with polyester knit fabric overlay. Channels, extending from the forefoot to heel regions, were filled with 100 % fluid United States Pharmacopeia ingestible grade glycerine. The flat (control) insoles were also constructed from ethylene-vinyl acetate plastic with polyester knit fabric overlay, but did not contain fluid-filled channels. These control insoles were used to isolate the effects of the glycerine-fluid on balance and walking, as there were no other differences in the composition or characteristics of the flat insoles relative to the glycerine-filled insoles. The contoured prefabricated foot orthoses investigated in this study (Orthaheel, Triplanar motion control, Vionic Group LLC, San Rafael, CA 94903, USA) were selected from a range of popular, commercially available inserts, designed for providing motion control. All the inserts were available in nine shoe sizes ranging from a women’s size 4.5 to men’s size 16 (United States sizing). The insoles and orthoses were worn within participants’ own closed shoes and were fitted by the investigators after removing the original footwear inserts.

### Outcome measures

Force data during the StandEO task was collected using a Kistler force platform (Model 9296AA, Kistler, Alton, UK). Data were sampled at 1,000 Hz (Power1401 Data Acquisition System, Cambridge Electronic Design, UK) and low-pass filtered (20 Hz, 4th order Butterworth filter) off-line using Matlab (The Mathworks, Nathick, USA). Balance measures during StandEO included CoP total path velocity (mm s^−1^), and the range and standard deviation of movement in mediolateral (ML Range, MLSD) and anterior-posterior (AP Range, APSD) directions (mm), and were analysed over the first 29 s of the task. CoP path velocity (mm s^−1^) represents the speed at which the centre of mass is moving, with a higher value indicating more rapid and potentially unstable movement [32]. CoP range (mm) is the maximum range of movement during the task in each ML and AP axes, with higher values indicating greater sway [32]. CoP SD (mm) corresponds to the variability about the average position of the CoP coordinate, with higher values suggesting greater exploratory (or less controlled) behaviour and movement patterns which are not constrained [33]. For the SEBT, reach distance in each of eight directions was measured in cm. Temporospatial gait parameters, measured using the GAITRite® system, were overall walking velocity (cm s^−1^), cadence (steps min^−1^), step length (cm), base of support (cm), double-limb support time (% gait cycle), toe in/out angle (°). After completing all balance and gait tasks for one insert, participants were asked to rate their perceived level of comfort on a 100 mm visual analogue scale (with 0 mm being extremely uncomfortable and 100 mm being extremely comfortable) [34]. Upon completion of all test procedures, participants were then asked to rank the relative comfort of each of the three inserts, from 1^st^ to 3^rd^ with 1^st^ being most comfortable and 3^rd^ being least comfortable. The three inserts were listed vertically and displayed in a randomised order for each participant. Both the ranking and rating scales have been previously validated and are reported to be reliable measures of footwear comfort [34].

### Statistical analysis

Statistical analyses were performed using SPSS version 22 (SPSS Inc, Chicago, IL 60606, USA). Data were examined for normality and homogeneity of variance. Three independent repeated measures multivariate analyses (MANOVA) were performed to determine any between-condition differences in CoP measures during StandEO, SEBT performance, and temporospatial gait parameters. The within-subjects factor was insert condition (glycerine-filled insole, prefabricated orthosis, flat insole). The dependent variables were CoP movement (CoP velocity, ML range, MLSD, AP range, APSD); SEBT reach distance (in anterior, anteromedial, medial, posteromedial, posterior, posterolateral, lateral, anterolateral directions); and temporospatial gait parameters (velocity, cadence, step length, base of support, double-limb support, toe in/out). Where a significant multivariate effect was observed, follow-up univariate tests (ANOVA) were performed. Repeated measures ANOVA were used to explore any between-condition differences in VAS rating scores for insert comfort. Where the assumption of sphericity was violated, a Greenhouse–Geisser correction was applied. Pearson’s correlation coefficients were used to explore whether VAS comfort rating scores were associated with CoP measures, SEBT reach distance, and temporospatial gait parameters for each of the insert conditions. A chi square test was used to determine any differences in the frequency with which each insert was ranked from most comfortable to least comfortable. The level of significance was set to 0.05, which was adjusted to 0.017 for each of the three MANOVAs.

## Results

### Single-leg standing with eyes open (StandEO)

Four participants were unable to successfully perform the StandEO without touching down with their raised leg, and were therefore excluded from the analyses. Repeated measures MANOVA showed no significant main effect of insert condition on CoP measures during StandEO (Wilks’ λ = 0.660, F (10,92) = 2.125, *P* = 0.030) (Table 1).

**Table 1:** Mean (± standard deviation) centre of pressure (CoP) movement during single-leg standing (*N* = 26)

CoP measure	Glycerine-Filled Insoles	Prefabricated Orthoses	Flat Insoles
ML Range (mm)	29.7 ± 3.6	30.6 ± 9.6	29.2 ± 3.3
MLSD (mm)	5.5 ± 0.7	5.5 ± 1.0	5.5 ± 0.7
AP Range (mm)	39.6 ± 10.4	36.8 ± 5.9	37.2 ± 6.4
APSD (mm)	7.2 ± 2.1	6.9 ± 1.3	7.0 ± 1.3
Velocity (mm s^−1^)	42.3 ± 10.0	40.0 ± 10.2	40.6 ± 9.1

### Star excursion balance test (SEBT)

Repeated measures MANOVA showed no significant between-condition differences for SEBT performance in any of the eight reach directions (Wilks’ λ = 0.644, F (16,102) = 1.572, *P* = 0.090) (Table 2).

**Table 2:** Mean (± standard deviation) reach distance during the star excursion balance test (SEBT) (*N* = 30)

SEBT Reach Direction	Glycerine-Filled Insoles	Prefabricated Orthoses	Flat Insoles
Anterior (cm)	83.7 ± 7.8	86.0 ± 8.7	85.0 ± 6.9
Anterolateral (cm)	85.0 ± 9.9	86.4 ± 9.9	86.7 ± 8.6
Lateral (cm)	82.6 ± 10.6	83.2 ± 9.2	83.5 ± 10.1
Posterolateral (cm)	78.7 ± 12.3	81.0 ± 11.2	82.3 ± 11.3
Posterior (cm)	78.0 ± 12.9	78.7 ± 11.2	79.3 ± 12.0
Posteromedial (cm)	74.2 ± 11.4	74.3 ± 9.1	73.6 ± 11.1
Medial (cm)	68.1 ± 11.0	67.4 ± 9.2	68.8 ± 11.5
Anteromedial (cm)	71.5 ± 9.1	73.1 ± 8.4	72.8 ± 9.4

### Level-ground walking

No significant multivariate effects of insole condition were observed for any temporospatial gait parameters during level ground walking (Wilks’ λ = 0.604, F (20,98) = 1.405, *P* = 0.138) (Table 3).

**Table 3:** Mean (± standard deviation) temporospatial gait measures during level-ground walking (*N* = 30)

Gait measure	Glycerine-Filled Insoles	Prefabricated Orthoses	Flat Insoles
Velocity (cm s^−1^)	136.4 ± 16.1	139.6 ± 14.3	138.7 ± 14.9
Cadence (steps min^−1^)	108.8 ± 8.2	110.0 ± 7.7	110.0 ± 8.0
Step Length (left leg) (cm)	75.1 ± 5.6	76.0 ± 5.5	75.5 ± 5.0
Step Length (right leg) (cm)	75.1 ± 5.3	76.2 ± 5.0	75.6 ± 4.7
Base of Support (left leg) (cm)	9.6 ± 2.5	9.4 ± 2.2	9.2 ± 2.7
Base of Support (right leg) (cm)	9.5 ± 2.6	9.3 ± 2.2	8.9 ± 3.3
Double-Limb Support Time (left leg) (% gait cycle)	23.0 ± 2.4	22.9 ± 2.3	22.7 ± 2.3
Double-Limb Support Time (right leg) (% gait cycle)	22.8 ± 2.4	22.9 ± 2.4	22.6 ± 2.4
Toe In/Out (left leg) (°)	2.5 ± 4.8	3.0 ± 4.5	2.5 ± 4.7
Toe In/Out (right leg) (°)	4.3 ± 4.9	4.5 ± 5.0	4.5 ± 4.5

### Perceived comfort

**Fig. 1:**
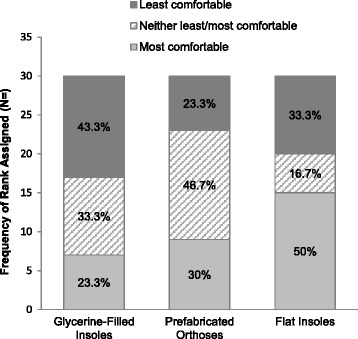
Perceived comfort ranking scores for the shoe inserts. Number and percentage of participants who ranked each insert from least to most comfortable, upon completion of all test procedures

Frequency analyses indicated that the flat insoles were most commonly ranked as the ‘most comfortable’ condition (*N* = 15), with the glycerine-filled insole most commonly reported to be ‘least comfortable’ (*N* = 13) (Fig. 1). Chi square test indicated that differences in comfort ranking scores between the three insert conditions were statistically significant (*χ*^2^ [4, *N* = 30] = 10.600, *P* = 0.031). Mean (SD) VAS comfort rating scores were 53.4 (23.2) mm (flat, control insole), 48.2 (23.6) mm (glycerine-filled insole) and 51.8 (24.7) mm (prefabricated orthosis), respectively. Repeated measures ANOVA reported no significant between-condition differences in VAS comfort ratings (F (2,58) = 0.453, *P* = 0.638). A significant, but weak correlation was observed between VAS rating scores for the flat insole with double-limb support time (left leg) during level-ground walking (*r* = −0.398, *P* = 0.029). Similarly, weak to moderate associations were reported between the prefabricated orthosis and posterior reach distance during the SEBT (*r* = −0.406, *P* = 0.026) and toe in/out angle during walking (*r* = −0.368, *P* = 0.045).

## Discussion

The primary aim of this study was to investigate the immediate effects of wearing novel glycerine-filled shoe insoles, contoured prefabricated orthoses, and a flat insole in healthy adults during standing balance tasks and level ground walking. We also explored any differences in perceived comfort between the three inserts. In contrast to our hypotheses, wearing glycerine-filled shoe insoles for the first time did not alter CoP movement during single-leg standing, SEBT performance, or temporospatial gait parameters during level-ground walking in asymptomatic adults, when compared to prefabricated foot orthoses or flat insoles. Therefore, future research is warranted, by way of proof of concept studies, to determine whether soft, fluid insoles, of varying thickness and viscosity, alter balance or walking performance, and to establish whether a threshold exists in these design features, at which an effect is observed.

Upon completion of all test procedures, the glycerine-filled insoles were most frequently ranked by participants as being the least comfortable. It is possible that this perceived level of comfort may reflect unfamiliarity to a new source of plantar sensory stimuli or the fluid foot-shoe interface, to which the participants were not accustomed. Although the participants were neither acclimatised to the prefabricated orthoses or flat insoles, the solid surface, material composition and density of these two inserts, is similar to the fabrication within usual footwear. Recent studies have observed an initial deterioration in balance or gait measures after short-term exposure to an unfamiliar source of plantar stimulation, including contoured dynamic foot orthoses [35] and textured shoe insoles [14, 36], in clinical and ageing populations. The flat insole was most frequently ranked as being the most comfortable condition. FPI scores indicated that normal foot posture was observed in 66.7 % of participants, with the remainder presenting differing degrees of pronation. This indicates that the majority of participants did not require biomechanical support or correction at the foot. Therefore, it is likely that the flat insole would best accommodate a normal foot position and facilitate movement in the path of least resistance [6], and as such, be perceived as the most comfortable condition. This is an important finding as the comfort of a footwear intervention is emerging as an important measure which appears to be closely associated with kinetic and kinematic responses [37, 38]. Notwithstanding these previous findings, the relationship between perceived comfort scores and biomechanical or clinical effectiveness requires further investigation.

Increased stability of the foot is one factor which has been proposed to contribute to greater perceived orthotic comfort [6]. This suggests that a contoured orthosis, which is moulded to and supports the arches of the foot, may be rated more comfortable than a flat insole. In the current study we observed no significant differences in VAS comfort rating scales between the three inserts investigated. These findings are in agreement with McPoil et al. [39] who observed no significant differences in VAS comfort scores between contoured orthoses and flat insoles following long-term wear, in people with and without patellofemoral pain. Furthermore, Mills et al. [37] reported that healthy adults rated a soft-flat, full-length orthosis to be more comfortable than three variations of contoured orthoses, which differed only in their degree of hardness. As the current work investigated asymptomatic adults who did not require orthotic prescription for biomechanical correction, or symptom control such as pain relief, this may explain why no clear distinction in level of comfort was experienced between the contoured and non-contoured conditions.

VAS comfort ratings for the flat control insoles were shown to be weakly associated with double-limb support time during walking. Perceived comfort ratings for the prefabricated orthosis were correlated to SEBT posterior reach distance and toe angle during ambulation. No significant correlations were identified between VAS comfort ratings for the glycerine-filled insoles and any of the outcome measures of interest. As only 3 significant, yet weak to moderate, correlations were observed out of a possible 84, these findings should be interpreted with caution as they provide only a suggestion that balance and walking performance may be associated with perceived comfort of shoe inserts. It is possible such correlations may be stronger in symptomatic populations. Indeed, previous research has shown that orthotic comfort may be related to underlying balance control mechanisms, specifically neuromuscular control, during challenging dynamic tasks [38, 40]. Alterations in lower limb neuromuscular function, including greater hip abduction and increased vastus lateralis activity whilst jogging [38] have been reported by adults with lower limb musculoskeletal conditions when wearing orthoses perceived to be less than comfortable. Such neuromuscular changes could represent compensatory strategies to maintain upright balance or optimal movement patterns, when wearing footwear interventions perceived to be uncomfortable. The potential impact of insert comfort (measured by way of visual analogue scales) on balance or gait performance may become more apparent when investigating neuromuscular mechanisms through which postural control is regulated.

### Study limitations

There were several limitations to this study. Firstly, a young asymptomatic population was explored, therefore our findings cannot be generalised to symptomatic clinical populations who may wear insoles or orthoses for therapeutic benefits. We felt it was important to explore the immediate effects of a novel glycerine-filled insole on balance and walking in healthy adults first in order to establish any potentially detrimental effects before applying them to patients with known balance impairments for whom insoles might be a helpful component of their management. Secondly, the viscous glycerine content of the novel insert is less likely to perturb the measures of single-leg standing, controlled voluntary movement during the SEBT, or level-ground walking at a comfortable pace that we studied, when compared to more dynamic activities such as reactive stepping, running, or jumping. Our findings cannot be extrapolated to these more dynamic activities before further research is conducted. Thirdly, due to obvious differences in the composition, structure, and feel of the inserts it was not possible to blind participants from the interventions under investigation. We countered potentially deleterious effects of non-blinding by keeping our participants unaware of the inserts of greatest interest in this study. Finally, participants wore their own footwear during test procedures, in order to reflect usual conditions to which they were already accustomed. However, it is possible that there may have been an interaction between the inserts and shoes.

## Conclusion

This study has demonstrated that a shoe insole, comprising fluid glycerine, does not alter single-leg balance performance or level-ground walking patterns, relative to wearing prefabricated orthoses or flat insoles, in healthy adults. These findings are specific to an asymptomatic population and as such further research is needed to determine the effects of glycerine-filled insoles in patients with balance impairments. Whilst the glycerine-filled insoles were most frequently ranked as being the least comfortable footwear condition, this may reflect immediate exposure to an unfamiliar source of sensory stimulation or unstable foot-shoe interface. Further investigation of the relationship between the perceived comfort of novel shoe insoles, balance and gait measures, including underlying neuromuscular mechanisms, is warranted.
